# A Novel Cross-Domain Mechanical Fault Diagnosis Method Fusing Acoustic and Vibration Signals by Vision Transformer

**DOI:** 10.3390/s24165120

**Published:** 2024-08-07

**Authors:** Zhenyun Chu, Shuo Xing, Baokun Han, Jinrui Wang

**Affiliations:** College of Mechanical and Electronic Engineering, Shandong University of Science and Technology, Qingdao 266590, China; chuzhenyun@sdust.edu.cn (Z.C.); bk_han@163.com (B.H.); wangjr33@163.com (J.W.)

**Keywords:** fault diagnosis, vibration signal, acoustic signal, parallel networks, fused features

## Abstract

Changes in operating conditions often cause the distribution of signal features to shift during the bearing fault diagnosis process, which will result in reduced diagnostic accuracy of the model. Therefore, this paper proposes a dual-channel parallel adversarial network (DPAN) based on vision transformer, which extracts features from acoustic and vibration signals through parallel networks and enhances feature robustness through adversarial training during the feature fusion process. In addition, the Wasserstein distance is used to reduce domain differences in the fused features, thereby enhancing the network’s generalization ability. Two sets of bearing fault diagnosis experiments were conducted to validate the effectiveness of the proposed method. The experimental results show that the proposed method achieves higher diagnostic accuracy compared to other methods. The diagnostic accuracy of the proposed method can exceed 98%.

## 1. Introduction

As the main transmission component of the servo motor, the health status of the bearing is directly related to the normal operation of the motor [1,2,3]. Therefore, it is essential to conduct fault diagnosis for servo motor bearings. Scholars have conducted extensive research on bearing fault diagnosis methods [4,5]. Zhang et al. [6] proposed a CNN-based method for converting raw signals into two-dimensional images for automatic feature extraction and fault diagnosis, improving accuracy and eliminating dependence on expert experience. Luo et al. [7] presented a diagnostic method using wavelet transform and neuro-fuzzy classification to reliably identify localized defects in ball bearings under varying load conditions. Wang et al. [8] proposes a novel machinery fault diagnosis approach using statistical locally linear embedding (S-LLE) for feature extraction and dimensionality reduction, significantly improving the classification performance of fault pattern recognition. Rai et al. [9] demonstrate the effectiveness of using a frequency domain approach with Hilbert–Huang transform (HHT) for bearing fault diagnosis, addressing inefficiencies in discrete Fourier transform (DFT) and wavelet transform (WT) methods. Kankar et al. [10] presented a wavelet-based feature extraction method using the minimum Shannon entropy criterion for diagnosing localized defects in ball bearings, demonstrating that support vector machines outperform other AI techniques in fault classification accuracy. Jia et al. [11] proposed a novel diagnostic method using S-transform, attention modules, and the Swin transformer framework to enhance fault feature detection in electronic circuits, achieving over 97% accuracy. Wang et al. [12] proposed a lightweight damage identification framework using optimized extreme learning machine and chaos game optimization to improve efficiency and accuracy in structural health monitoring systems. Although these methods are able to achieve relatively good fault diagnosis for rolling bearings, they all suffer from reduced generalization ability and poor robustness when the diagnostic scenario changes.

In recent years, transfer learning theory has been gradually developed. This provides a new approach to improving the generalization capability of fault diagnosis methods. Wang et al. [13] believe domain adaptation techniques, particularly domain-adversarial neural networks (DANNs), are promising for improving the generalization of fault diagnosis models across different machines in a fleet, reducing the need for manual data labeling and model modification. Gou et al. [14] proposed a deep convolutional transfer learning network (DCTLN) that effectively addresses the challenges of intelligent fault diagnosis for machines with unlabeled data by combining condition recognition through a 1D CNN and domain adaptation to learn domain-invariant features. Han et al. [15] addressed the challenge of machinery fault diagnosis with sparse target data by pairing source and target data under the same conditions for individual domain adaptation. Wu et al. [16] proposed a few-shot transfer learning method using meta-learning to improve rotating machinery fault diagnosis under variable conditions with scarce fault samples, demonstrating its effectiveness across multiple datasets and transfer scenarios. Liu et al. [17] demonstrated the effectiveness of a transfer-learning-based methodology for improving fault diagnosis in building chillers, particularly in data-sparse scenarios, achieving significant accuracy improvements in experimental results. Wang et al. [18] proposed a multi-scale deep intra-class adaptation network for machine fault diagnosis, effectively addressing varying working conditions and data scarcity, validated by high-precision results in multiple transfer learning experiments. Wen et al. [19] introduced TranVGG-19, a transfer learning approach utilizing pre-trained VGG-19 for fault diagnosis, achieving 99.175% accuracy and rapid training on motor bearing data by converting time-domain signals to RGB images for feature extraction. Zhu et al. [20] presented a new fault diagnosis method that extends convolutional neural networks (CNNs) to transfer learning scenarios, enhancing model adaptability and reducing distribution discrepancies through layer-wise adaptation and multi-kernel domain loss, achieving superior fault classification performance. Lee et al. [21] proposed new metrics and domain alignment techniques to improve transfer learning for bearing fault diagnosis across different operating speeds and specifications, achieving better performance even with limited data. These researchers have utilized transfer learning theory for fault diagnosis and achieved excellent results. However, the diagnostic accuracy of these methods still needs improvement.

To address the issue of low diagnostic accuracy under certain operating conditions, researchers have proposed using multi-sensor signals for fault diagnosis. Kumar et al. [22] presented a low-cost, multi-sensor data acquisition system using an Arduino micro-controller to detect faults in FDM-based 3D-printed products, achieving around 94% accuracy with a CNN model. Dong et al. [23] introduced an integrated multi-sensor diagnosis and prognosis platform using a hidden semi-Markov model (HSMM), validated on Caterpillar Inc. hydraulic pumps, showing promising results in improving diagnostic accuracy and enabling equipment prognosis. Tang et al. [24] presented an expert system using multi-sensor data fusion to enhance the real-time machining accuracy of thin-walled lens barrels, achieving high predictive accuracy for material removal rates, tool status, and chip status, thus improving efficiency and yield in optical lens production. Guan et al. [25] proposed a multi-sensor and multi-scale model (2MNet) for accurate fault diagnosis of rolling bearings, integrating multi-directional vibration signals and advanced feature extraction techniques to enhance the reliability of rotating machinery. Sun et al. [26] proposed a multi-sensor fusion convolutional neural network (MF-CNN) that combines vibration and sound signals for enhanced fault diagnosis in rotating machinery, demonstrating superior classification accuracy and robustness in both rolling bearing and gas turbine applications. However, these methods still have the following issues: 1. The ability to directly capture long-range dependencies in time series signals needs improvement. 2. There is a lack of capability to globally weigh and focus on important time-step features of acoustic and vibration signals, thereby effectively identifying long-term trends and patterns in the signals. 3. The accuracy of cross-domain fault diagnosis through the fusion of acoustic and vibration signal features still needs improvement. Therefore, this paper proposes a dual-channel parallel adversarial network for bearing fault diagnosis through the fusion of acoustic and vibration signal features. The main contributions of this paper are as follows:

(1) A unique parallel network is constructed using vision transformer blocks. This network is employed to achieve the extraction and fusion of acoustic and vibration signal features.
(2) The generalization of the fused features of acoustic and vibration signals is improved through the combined approach of adversarial training and Wasserstein distance metrics.

The latter four parts of this paper are, respectively: Theoretical Background, Framework of DPAN, Experimental Validation, and Conclusion. In the Theoretical Background section, unsupervised domain adaptation and self-attention mechanisms are introduced. In the DPAN framework section, the structural information of the proposed method is presented. Finally, the proposed method is validated and conclusions are drawn through two sets of cross-domain fault diagnosis experiments.

## 2. Theoretical Background

### 2.1. Unsupervised Domain Adaptation

**Figure 1:**
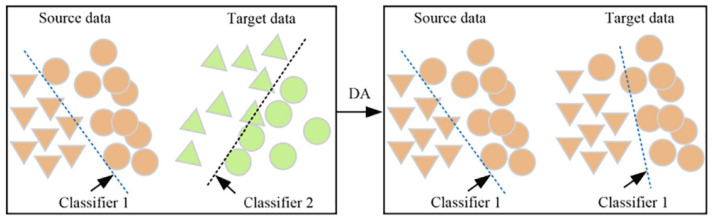
The process of domain adaptation [27].

Traditional machine learning is based on the assumption that, when solving feature classification problems, training samples and test samples come from the same distribution. However, in practical applications, many factors can lead to different data distributions, and collecting enough supervised data to train the network involves a huge amount of work. This issue can be addressed by transferring the knowledge from supervised data to unsupervised data through transfer learning. However, due to the inconsistency in data distribution, network performance can degrade. Domain adaptation aims to solve this problem by adjusting the differences between domains. The domain adaptation process is shown in Figure 1 [27]. Currently, there are three mainstream domain adaptation methods: (1) discrepancy-based methods, (2) adversarial methods, and (3) reconstruction-based methods. This paper employs the first two methods. Discrepancy-based methods: these methods align the feature distributions of different domains by minimizing the distributional differences between them. Depending on the measurement and transformation approaches, discrepancy-based methods can be further divided into statistical transformation, structural optimization, and geometric transformation sub-methods. Unlike DA, UDA does not rely on the labels of the target domain samples. Therefore, unsupervised domain adaptation is able to find common features from labeled source domain data and unlabeled target domain data, thereby achieving cross-domain feature extraction.

### 2.2. Self-Attention Mechanism

**Figure 2:**
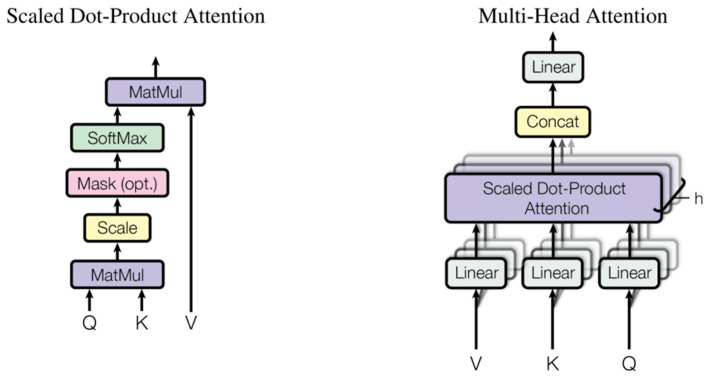
Multi-headed attention mechanism [28].

The self-attention mechanism is a technique in deep learning that calculates the similarity between different positions in a sequence, generates attention weights based on these similarities, and then computes a weighted sum to obtain the final representation for each position. This effectively captures long-range dependencies in sequence data. The structure of the self-attention mechanism is shown in Figure 2 [28]. This mechanism finds wide applications in computer vision and natural language processing, such as in transformer models, to enhance model performance in handling complex tasks. As a representative self-attention mechanism, the structure and formulas of the multi-head attention mechanism are as follows:

(1) $$\mathrm{Attention}\left(Q,K,V\right)=\;\mathrm{softmax}\left(\frac{QK^{T}}{\sqrt{d_{k}}}\right)V$$

where *Q*, *K*, and *V* represent query, key, and value, respectively. They play crucial roles in the process of computing attention weights and performing the weighted sum.

## 3. Dual-Channel Parallel Adversarial Network (DPAN)

The proposed dual-channel parallel adversarial network mainly consists of five components: a parallel feature extractor based on vision transformer, an acoustic–vibration signal fusion feature extraction module, a distance metric module, a fault classification module, and a domain classification module. The structure of DPAN is as follows:

- Step 1: The time-domain signals of acoustics and vibrations are segmented and transformed into frequency-domain signals through FFT.
- Step 2: The frequency-domain signal datasets of acoustics and vibrations are fed into the network, then segmented again to a certain window size and encoded.

(2) $$z_{0}=\mathrm{Linear}\;\mathrm{projection}\left[x_{p}^{1};x_{p}^{2};\ldots .;x_{p}^{N}\right]$$

where *x* is the patch after signal segmentation, $z_{0}$ is patch embedding.

- Step 3: The encoded frequency-domain signals of acoustics and vibrations are fed into parallel feature extractors for separate feature extraction.
- Step 4: The acoustic and vibration signal features are fused using a self-attention mechanism and measured by the Wasserstein distance.

(3) $$W\left(P_{r},P_{g}\right)=\underset{\gamma \in \prod \left(P_{r},P_{g}\right)}{\inf}{Ε}_{\left(x,y\right)}\left[∥x-y∥\right]$$

- Step 5: The fused features are fed separately into the fault classifier and domain classifier.

### 3.1. Dataset Processing

The acoustic and vibration time-domain signals are segmented into samples of length 2048, and then are transformed into frequency-domain signals. Since the transformed signals are symmetric, the first 1024 points of the frequency-domain signals are taken as the input samples for the network.

### 3.2. The Framework of DPAN

**Figure 3:**
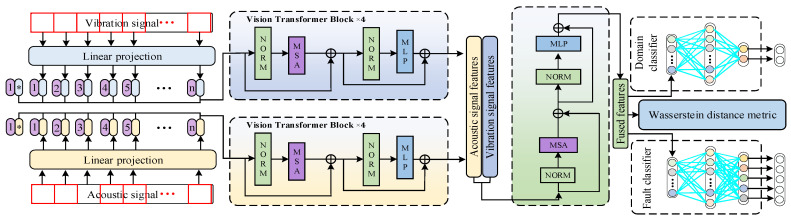
The framework of DPAN.

To obtain more feature information that can reflect the operating state of the bearing from acoustic signals and vibration signals, parallel feature extraction is performed on both types of signals. The fused features of acoustic and vibration signals are obtained through parallel feature extraction and fusion based on the self-attention mechanism. In this process, the parallel feature extraction network and the fusion module are utilized. The framework of DPAN is displayed in Figure 3.

First, before parallel feature extraction is performed, the fused features are segmented and embedded to obtain patches. Specifically, this process can be achieved through a CNN. The paradigm of the embedding process is as follows:

(4) $$P=\left({\left(\underset{k}{\sum }x_{k}^{l-1}\times w_{n}^{l}+b_{n}^{l}\right)}_{j},x_{j}\right)$$

where P represents the encoded patch, k represents the size of the segmentation window, and $x_{j}$ is the *j*-th point of the input signal segment.

Then, the patches of the acoustic signals and vibration signals are separately fed into two parallel feature extraction networks. During this process, the features of the acoustic signals and vibration signals are extracted independently. The independent feature extraction method can prevent interference between the features of the acoustic signals and vibration signals. As the network deepens, the representative features are gradually obtained. The experiments conducted in this paper used four vision transformer blocks on the channels for feature extraction of acoustic and vibration signals, respectively.

(5) $$f_{l}^{\prime }=MSA\left(LN\left(f_{l-1}\right)\right)+f_{l-1}$$

(6) $$f_{l}=MLP\left(LN\left(f_{l}^{\prime }\right)\right)+f_{l}^{\prime }$$

where $f_{l}$ is the output of the *l*-th vision transformer block.

After being processed by parallel feature extraction, the acoustic and vibration signals are concatenated for feature fusion. The features are fused through an attention mechanism based on the vision transformer. During this process, the redundant features are effectively suppressed, while the informative features are emphasized. Subsequently, the fused features are measured by the Wasserstein distance, thereby obtaining more domain-invariant features during the training process. The definition of the Wasserstein distance loss is as follows:

(7) $$L_{wd}=\left|\frac{1}{n^{s}}\overset{n^{s}}{\underset{i=1}{\sum }}h_{i}^{s}-\frac{1}{n^{t}}\overset{n^{t}}{\underset{i=1}{\sum }}h_{j}^{t}\right|$$

where $h_{i}^{s},\;h_{j}^{t}$ denote the features of the different domains.

Finally, the fused features are separately input into a label classifier and a domain classifier. A gradient reversal layer is placed at the connection between the feature extractor and the domain classifier to facilitate adversarial training. The label classification loss and domain classification loss are as follows:

(8) $$L_{C}\left(x^{s},y^{s}\right)=\frac{1}{n}\sum_{i=1}^{n^{s}}\sum_{k=1}^{K}l\left(y_{i}^{s}=k\right)\cdot logC{\left(F\left(x_{i}^{s}\right)\right)}_{k}$$

where $l\left(y_{i}^{s}=k\right)$ corresponds to the indicator function, $x^{s}$ are the predicted values, and $n^{s}$ is the number of samples in the source domain.

(9) $$L_{D}=\frac{1}{n^{s}}\overset{n^{s}}{\underset{i=1}{\sum }}{Ε}_{x^{s}∼p_{r}}D(F(x_{i}^{s}))+\frac{1}{n^{t}}\overset{n^{t}}{\underset{j=1}{\sum }}{Ε}_{x^{t}∼P_{g}}D\left(F\left(x_{j}^{t}\right)\right)\;$$

### 3.3. Model Training

The model is trained using the Adam optimizer. During the training process, the parameters of the feature extractor $\theta_{F}$, domain classifier $\theta_{D}$, and label classifier $\theta_{C}$ are optimized separately. Ultimately, the best parameters for the model are obtained.

The total loss function of the model DPAN is as follows:

(10) $$L\left(\theta_{F},\theta_{C},\theta_{D}\right)=\gamma L_{C}\left(\theta_{F},\theta_{C}\right)+\beta L_{wd}\left(\theta_{F}\right)-\lambda L_{D}\left(\theta_{F},\theta_{D}\right)$$

The parameter optimization process for DPAN is as follows:

(11) $$\theta_{F}\leftarrow \theta_{F}-\alpha \left(\gamma \frac{\delta L_{C}}{\delta \theta_{F}}+\beta \frac{\delta L_{wd}}{\delta \theta_{F}}-\lambda \frac{\delta L_{D}}{\delta \theta_{F}}\right)$$

(12) $$\theta_{C}\leftarrow \theta_{C}-\alpha \left(\gamma \frac{\delta L_{c}}{\delta \theta_{C}}\right)$$

(13) $$\theta_{D}\leftarrow \theta_{D}-\alpha (\lambda \frac{\delta L_{D}}{\delta \theta_{D}})$$

where α represents the learning rate and γ,β,λ are the weighting factors of the three losses.

### 3.4. Comments

The proposed method in this paper fuses acoustic and vibration signals through a vision-transformer-based parallel feature extraction and fusion network. The fused features are subjected to adversarial training and feature domain distance reduction, which allows the network’s cross-domain fault diagnosis capability to be improved. Therefore, the method proposed in this paper should be applied to diagnostic scenarios where the source domain conditions contain labels and the target domain conditions are unlabeled.

## 4. Experimental Verification

### 4.1. Data Description

**Figure 4:**
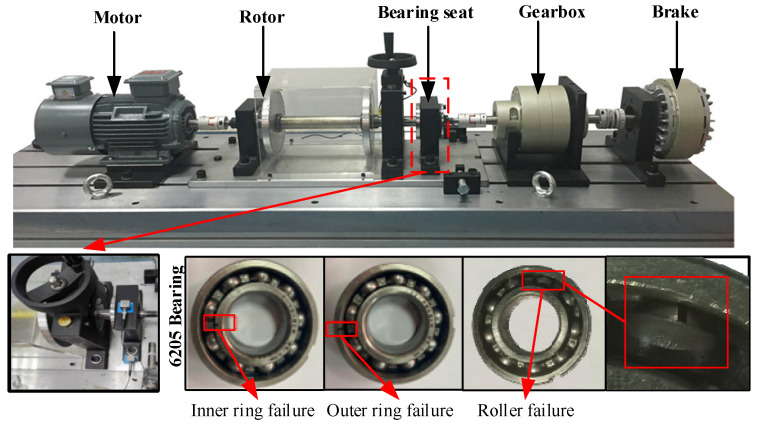
Experimental platform and faulty bearing diagram.

This paper uses the rotating machinery fault diagnosis experimental platform of Shandong University of Science and Technology [29] to collect acoustic and vibration signals. The faulty bearing is a 6205 deep groove ball bearing. The experimental platform and bearing photos are shown in Figure 4. During the experiment, the test bench was set with six different operating conditions, including three different speeds and three different loads. The three speeds are, respectively: 1300 rpm (A1), 1800 rpm (A2), 2200 rpm (A3). The three loads are, respectively: 0 N (S1), 20 N (S2), 60 N (S3). It is worth noting that the signals under the three different loads were all collected when the bearing speed was 1800 rpm. The bearings used in the experiment are set with five types of faults: inner ring failure 0.2 mm (IF0.2), outer ring failure 0.2 mm (OF0.2), rolling element failure 0.2 mm (RF0.2), outer ring and rolling element mixed fault (ORF0.2), and normal condition bearing.

### 4.2. Experiment 1: Bearing Fault Diagnosis Experiment under Different Speeds

The diagnostic results of the proposed method are compared with the diagnostic results of three state-of-the-art transfer learning methods in order to validate the effectiveness of the proposed method. Experiment 1 set up six groups of transfer tasks, as detailed in Table 1.

Method 1: Transfer component analysis (TCA) [30].

Method 2: Feature-based transfer neural network (FTNN) [31].

Method 3: Distance-based deep transfer learning (WD-DTL) [32].

**Figure 5:**
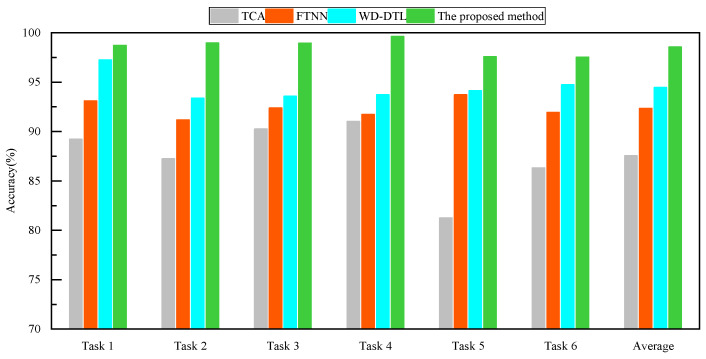
Bar chart of diagnostic accuracy in Experiment 1.

Table 2 and Figure 5 show the diagnostic results of the four methods in Experiment 1. It can be observed that TCA achieves a diagnostic accuracy of over 85% for all tasks. However, the diagnostic accuracy of TCA is significantly lower in Task 5 and Task 6 compared to other tasks. FTNN achieves a diagnostic accuracy of over 91% in Experiment 1. FTNN has higher diagnostic accuracy compared to TCA, and the diagnostic accuracies across the six transfer tasks are similar. Compared to the first two methods, WD-DTL performs better with an average diagnostic accuracy of 94%. Moreover, its diagnostic accuracy in Task 1 can exceed 97%. By observing the diagnostic results, it can be seen that DPAN has a higher diagnostic accuracy compared to the other three methods. The average diagnostic accuracy of DPAN can reach over 98%.

**Figure 6:**
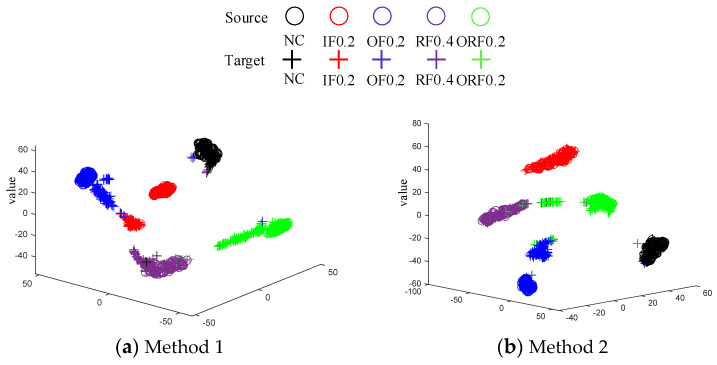

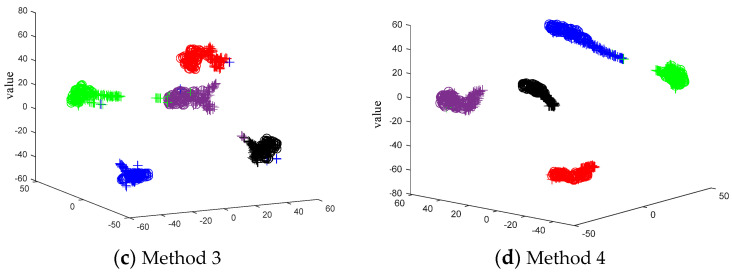
Dimensionality reduction graph of feature extractor outputs for the four methods in Experiment 1.

Figure 6 shows the t-SNE [33] dimensionality reduction plots of the feature extractor outputs for the four methods in Task 5. It can be observed that TCA fails to achieve correct clustering for many sample points, and the source and target domains of the IF and OF samples are difficult to align. By observing Figure 6b, it can be found that the domain alignment capability of FTNN is also insufficient. The distance between the source-domain sample clusters and the target-domain sample clusters in WD−DTL has decreased compared to the previous two methods. However, WD−DTL still fails to correctly cluster some sample points. Compared to the other three methods, the proposed method can effectively achieve domain alignment, and the sample clusters for the five types of faults do not show any overlapping.

**Figure 7:**
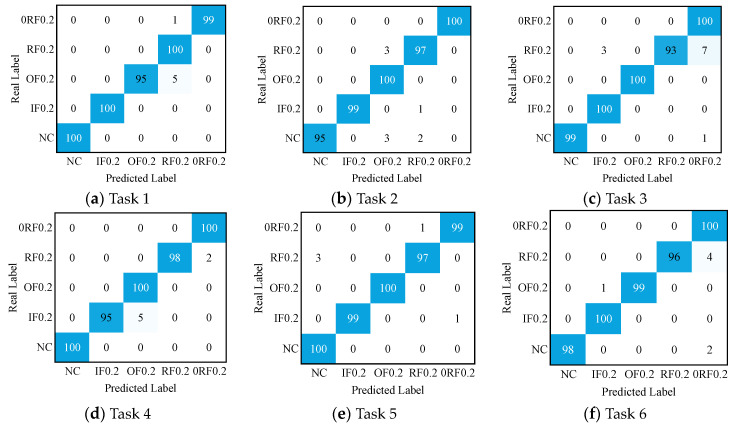
Confusion matrix of the diagnostic results for the six transfer tasks in Experiment 1.

Figure 7 shows the confusion matrix of the diagnostic results for DPAN in the six transfer tasks. It can be observed that DPAN achieves precise classification for most of the fault signals. It is worth noting that DPAN still exhibits misclassification in a few tasks. For example, in Task 1 and Task 3, DPAN exhibits misclassification for OF. In Tasks 2, 3, 4, and 5, the ability to recognize IF and RF needs improvement.

### 4.3. Experiment 2: Bearing Fault Diagnosis Experiment under Different Loads

After conducting transfer experiments under different speed conditions, this paper carried out cross-domain fault diagnosis experiments under different load conditions. The setup for the transfer tasks is shown in Table 3.

**Figure 8:**
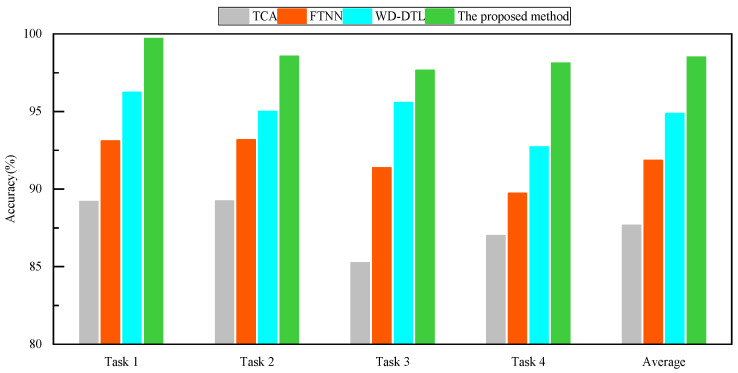
Bar chart of diagnostic accuracy in Experiment 2.

**Figure 9:**
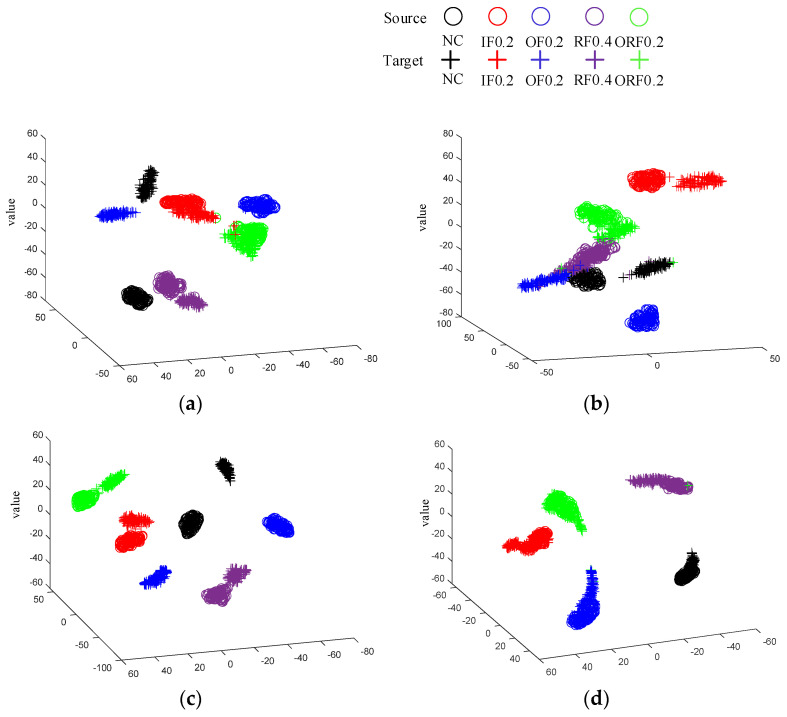
Feature dimension reduction diagrams for each stage. (**a**) The vibration signals after feature extraction. (**b**) The acoustic signals after feature extraction. (**c**) The fused features of the equal−weight fusion method. (**d**) The fused features of DPAN.

**Figure 10:**
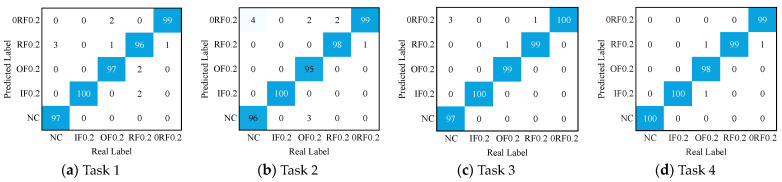
Confusion matrix of the diagnostic results for the four transfer tasks in Experiment 2.

Table 4 and Figure 8 show the diagnostic accuracy of the four methods in Experiment 2. It is easy to observe that, corresponding to Experiment 1, the proposed DPAN achieves the highest diagnostic accuracy in the four transfer tasks compared to the other three methods. Figure 9 shows the feature dimensionality reduction maps at different stages of the network, as well as the dimensionality reduction maps of the fused features obtained by training DPAN with the common equal−weight fusion method in Task 3. It can be observed that the acoustic and vibration signals after feature extraction already show a clustering trend. Subsequently, through feature fusion, the various sample clusters exhibit good clustering and alignment. Additionally, by comparing Figure 9b and Figure 9d, it can be seen that the proposed feature fusion method is more conducive to sample clustering and domain alignment. Figure 10 shows the confusion matrix of the diagnostic accuracy for DPAN in the four tasks of Experiment 2. By observing Figure 10, it can be seen that the proposed method achieves high classification accuracy for various fault samples.

Although the proposed method has a superior performance in the two sets of experiments, the problem of degraded diagnostic accuracy still occurs in fault diagnosis scenarios with large domain differences. For example, in the transfer task with different rotational speeds, the diagnostic accuracy of the proposed method in Task 5 and Task 6 is obviously lower than that of the other methods.

## 5. Conclusions

To address the issue of reduced diagnostic accuracy caused by changes in operating conditions and the simplification of network input information, this paper proposes a DPAN. In the feature extraction process, acoustic and vibration signals are extracted in parallel, and a self-attention mechanism is used to fuse the features of acoustic and vibration signals. Then, the fused features of the acoustic and vibration signals are measured using the Wasserstein distance. Finally, the capability to extract domain-invariant fused features is enhanced by performing adversarial training on the network. In the experiments, the diagnostic accuracy of the proposed method can reach more than 98%, which indicates that the proposed method has good implementability in the cross-speed fault diagnosis condition and cross-load fault diagnosis condition. In addition, during the experiments, the proposed method shows good domain alignment capability, which verifies that the proposed method can effectively extract the fusion features of acoustic and vibration signals across domains. There are shortcomings in the study under fluctuating speed conditions. We will research the bearing fault diagnosis method under fluctuating speed conditions in the next phase. Moreover, in actual working scenarios, acoustic signals and vibration signals are often subjected to a lot of noise interference. Therefore, how to effectively reduce the impact of environmental noise on diagnostic results should also be a key focus of future research.

## Figures and Tables

**Table 1:** Transfer tasks in Experiment 1.

	Task 1	Task 2	Task 3	Task 4	Task 5	Task 6
Transfer tasks	A1→A2	A2→A1	A2→A3	A3→A2	A1→A3	A3→A1

**Table 2:** Accuracy of fault diagnosis using four methods in Experiment 1.

Method	Task 1	Task 2	Task 3	Task 4	Task 5	Task 6	Average
Method 1	89.2%	87.23%	90.25%	91%%	81.24%	86.31%	87.538%
Method 2	93.1%	91.17%	92.37%	91.72%	93.71%	91.93%	92.333%
Method 3	97.23%	93.37%	93.57%	93.72%	94.12%	94.73%	94.456%
Proposed	98.71%	98.96%	98.95%	99.62%	97.56%	97.53%	98.555%

**Table 3:** Transfer tasks in Experiment 2.

	Task 1	Task 2	Task 3	Task 4
Transfer tasks	S1→S2	S2→S3	S2→S1	S3→S2

**Table 4:** Accuracy of fault diagnosis using four methods in Experiment 2.

Method	Task 1	Task 2	Task 3	Task 4	Average
Method 1	89.2%	89.27%	85.29%	87%	87.67%
Method 2	93.15%	93.27%	91.57%	89.62	91.85%
Method 3	96.26%	95.06%	95.47%	92.69%	94.68%
Proposed	99.71%	98.56%	97.65%	98.12%	98.51%

## Data Availability

The data can be accessed through the corresponding authors by reasonable request.
